# Evolution of enzymes with new specificity by high-throughput screening using DmpR-based genetic circuits and multiple flow cytometry rounds

**DOI:** 10.1038/s41598-018-20943-8

**Published:** 2018-02-08

**Authors:** Kil Koang Kwon, Dae-Hee Lee, Su Jin Kim, Su-Lim Choi, Eugene Rha, Soo-Jin Yeom, Bindu Subhadra, Jinhyuk Lee, Ki Jun Jeong, Seung-Goo Lee

**Affiliations:** 1 Synthetic Biology and Bioengineering Research Centre, Korea Research Institute of Bioscience and Biotechnology (KRIBB), Daejeon, 305-806 Korea; 2 Department of Chemical and Biomolecular Engineering, Korea Advanced Institute of Science and Technology (KAIST), Daejeon, 305-338 Korea; 3 Biosystems and Bioengineering Program, University of Science and Technology (UST), Daejeon, 305-350 Korea; 4 Korean Bioinformation Centre (KOBIC), Korea Research Institute of Bioscience and Biotechnology (KRIBB), Daejeon, 305-806 Korea; 5 Department of Bioinformatics, University of Sciences and Technology (UST), Daejeon, 305-350 Korea

## Abstract

Genetic circuit-based biosensors are useful in detecting target metabolites or *in vivo* enzymes using transcription factors (Tx) as a molecular switch to express reporter signals, such as cellular fluorescence and antibiotic resistance. Herein, a phenol-detecting Tx (DmpR) was employed as a critical tool for enzyme engineering, specifically for the rapid analysis of numerous mutants with multiple mutations at the active site of tryptophan-indole lyase (TIL, EC 4.1.99.1). Cellular fluorescence was monitored cell-by-cell using flow cytometry to detect the creation of phenolic compounds by a new tyrosine-phenol-lyase (TPL, EC 4.1.99.2). In the TIL scaffold, target amino acids near the indole ring (Asp^137^, Phe^304^, Val^394^, Ile^396^ and His^463^) were mutated randomly to construct a large diversity of specificity variations. Collection of candidate positives by cell sorting using flow cytometry and subsequent shuffling of beneficial mutations identified a critical hit with four mutations (D137P, F304D, V394L, and I396R) in the TIL sequence. The variant displayed one-thirteenth the level of TPL activity, compared with native TPLs, and completely lost the original TIL activity. The findings demonstrate that hypersensitive, Tx-based biosensors could be useful critically to generate new activity from a related template, which would alleviate the current burden to high-throughput screening.

## Introduction

The recent progress in synthetic biology has encouraged the synthesis of custom-made enzymes and directed the rapid evolution of new specificities^1–3^. The combined use of rational designs and random mutagenesis has been the preferred choice in most enzyme engineering studies^4,5^, and has resulted in the generation of a large genetic diversity that can easily exceed one million by the random mutagenesis of just 5–6 residues. Therefore, the practical problem at this stage is the limited throughput of the screening used to identify positives, which is usually too slow to cover the genetic diversity. These rapid profiling and screening issues have become more important with the rise of synthetic biology, which requires the optimisation of multiple amino acids to generate custom-made activities or specificities in target structures^6,7^.

The first step to generate a custom-made specificity is the selection of a scaffold protein with appropriate structures. In this study, we selected tryptophan indole-lyase (TIL, EC 4.1.99.1) as the template to evolve a new tyrosine phenol-lyase (TPL, EC 4.1.99.2). These two lyases naturally share tetrameric structures catalysing α, β-elimination and β-replacement of their own substrates and use pyridoxal-5′-phosphate (PLP) as the coenzyme^8,9^.

Next, the selection of target amino acids is important to construct a focused library that may create a large diversity of possible functions in the template. For example, the design of loop libraries for the metallo-hydrolase active site could result in the detection of non-natural β-lactamase activity after extensive screening for antibiotic resistance^10^. More recently, a novel enzyme termed formolase was created from a benzaldehyde lyase through a focused library design and robotic high-throughput screening^11^. Specificity engineering with *Citrobacter freundii* TPL sought to confer TIL activity by replacing the active site residues with the conserved residues in TILs. However, despite the high structural similarity between two scaffolds, this attempt was not successful because of the lack of a sensitive and high-throughput assay to test the diversities of the probable mutations^12^.

In the present study, using screening based on phenol-detecting transcription factors (Tx) we tested the large diversities of a focused library with multiple combinations of five mutations near the substrate site of TIL, which were targeted by the comparison of structural homologues and related sequences. The diversity libraries were analysed using flow cytometry, which enables cell sorting based on cellular fluorescence and which is increasingly being used for this sort of analysis^13–15^. The fluorescence signal is obtained from specific Tx triggering the expression of cellular fluorescence in response to specific metabolites or *in vivo* enzymes.

We recently reported that a phenol-detecting Tx (DmpR) termed the genetic enzyme-screening system (GESS) can detect numerous phenol-generating enzymes when combined with designed substrates^16^. More than 200 enzyme species were assumed to be detectable by the combinations of the GESS technique and specific phenol-generating substrates, referring to the BRENDA database^17^. This report demonstrates that the GESS is very appropriate as a throughput tool to test the creation of the α, β-elimination of l-tyrosine in the TIL active site.

## Results

**Figure 1:**
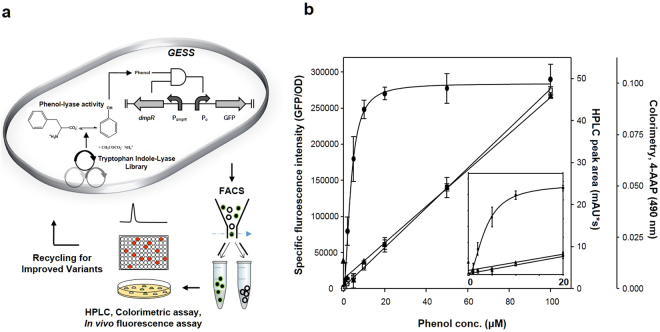
Genetic enzyme-screening principle for evolutionary enzyme engineering. (**a**) Genetic libraries were constructed by site-saturation mutagenesis and staggered extension process. A genetic enzyme-screening system (GESS) was used to screen the constructed libraries for extracting active cells that triggered the genetic circuit to express a fluorescent reporter protein in the presence of phenol-lyase. Active variants with fluorescence were verified by HPLC or colorimetric assay. (**b**) Comparison of relative sensitivity for phenol detection by GESS (⦁), HPLC (◼) or colorimetric assay (▴). Inside panel shows the enlarged version ranging from 0 to 20 μM phenol. Each experiment was performed in triplicate.

Figure 1a depicts the genetic circuit-based strategy for the development of novel enzyme activities. Saturation mutagenesis diversified the selected residues in the TIL scaffold followed by the expression of gene libraries in the host cells harbouring the biosensor circuit, pGESS^13^. In the appearance of phenolic products, DmpR was activated to express fluorescent proteins in the corresponding cells, which were collected using flow cytometry. The obtained cells were regrown and the activity was determined using high-performance liquid chromatography (HPLC) or colorimetric analyses (Fig. 1a). The sensitivity of the flow cytometric screening was remarkably higher, while the error rate was reasonable, relative to the conventional analytical methods (inner panel of Fig. 1b).

### Rational design and generation of *Escherichia coli* TIL mutant library

**Figure 2:**
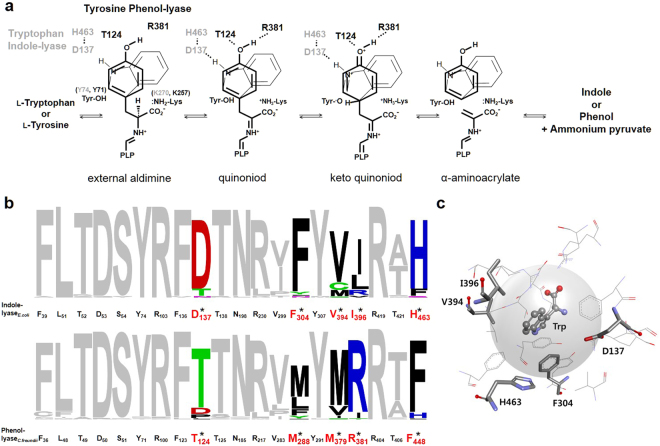
Target residues for the development of phenol-lyase activity from an indole-lyase scaffold. (**a**) Overlapping schemes of β-elimination of l-tyrosine and l-tryptophan catalysed by phenol-lyase (thick line and black letters) and indole-lyase (thin line and grey letters), respectively. (**b**) Conserved residues in the active sites of indole-lyase (upper) and phenol-lyase (lower) within a distance of 6 Å from the substrate. The y-axis represents the conservation of residues in each of 50 enzymes from the NCBI database. Asterisks indicate the characteristic residues conserved differently between indole-lyase and phenol-lyase. (**c**) Structural representation of five characteristic residues (Asp^137^, Phe^304^, Val^394^, Ile^396^, and His^463^) in tryptophan indole-lyase from *Escherichia coli* (PDB ID: 2C44).

*E. coli* TIL was used as the template, in this study, to create TPL activity. This TIL shares many catalytic residues and mechanisms with TPLs (Fig. 2a). The recruitment of critical residues from analogous structures has been a preferred approach to design new activities in similar templates, while maintaining the residues necessary for the conserved function^18,19^. This approach was examined for TPL of *Citrobacter freundii* by recruiting residues from TIL of *Proteus vulgaris*. However, this attempt did not lead to the development of TIL activity in the TPL template^12^.

In recognition of the prevalence of the genetic biosensor technology, we designed a wide diversity of mutants by selecting multiple residues to randomize within 6 Å from the substrate, referring to the conservation statistics (Fig. 2b) and three-dimensional structures of TIL (Fig. 2c). The conservation was compared with 50 sequences from the NCBI database and represented using a WebLogo diagram (Fig. 2b)^20^. The WebLogo diagram of the two lyases showed mostly analogous profiles, excluding five residues conserved differently in the two active sites. Asp^137^, Phe^304^, Val^394^, Ile^396^, and His^463^ were conserved as Thr^124^, Met^288^, Met^379^, Arg^381^, and Phe^448^ in the phenol-lyases, respectively (Fig. 2b). These five amino acids at the active site of *E. coli* TIL were selected as the target residues for saturation mutagenesis to induce promiscuity in substrate specificity. The library size was approximately 6 × 10^6^. The focused mutation was confirmed by sequencing 30 randomly selected mutants.

### Screening of *E. coli* TIL mutant library

**Figure 3:**
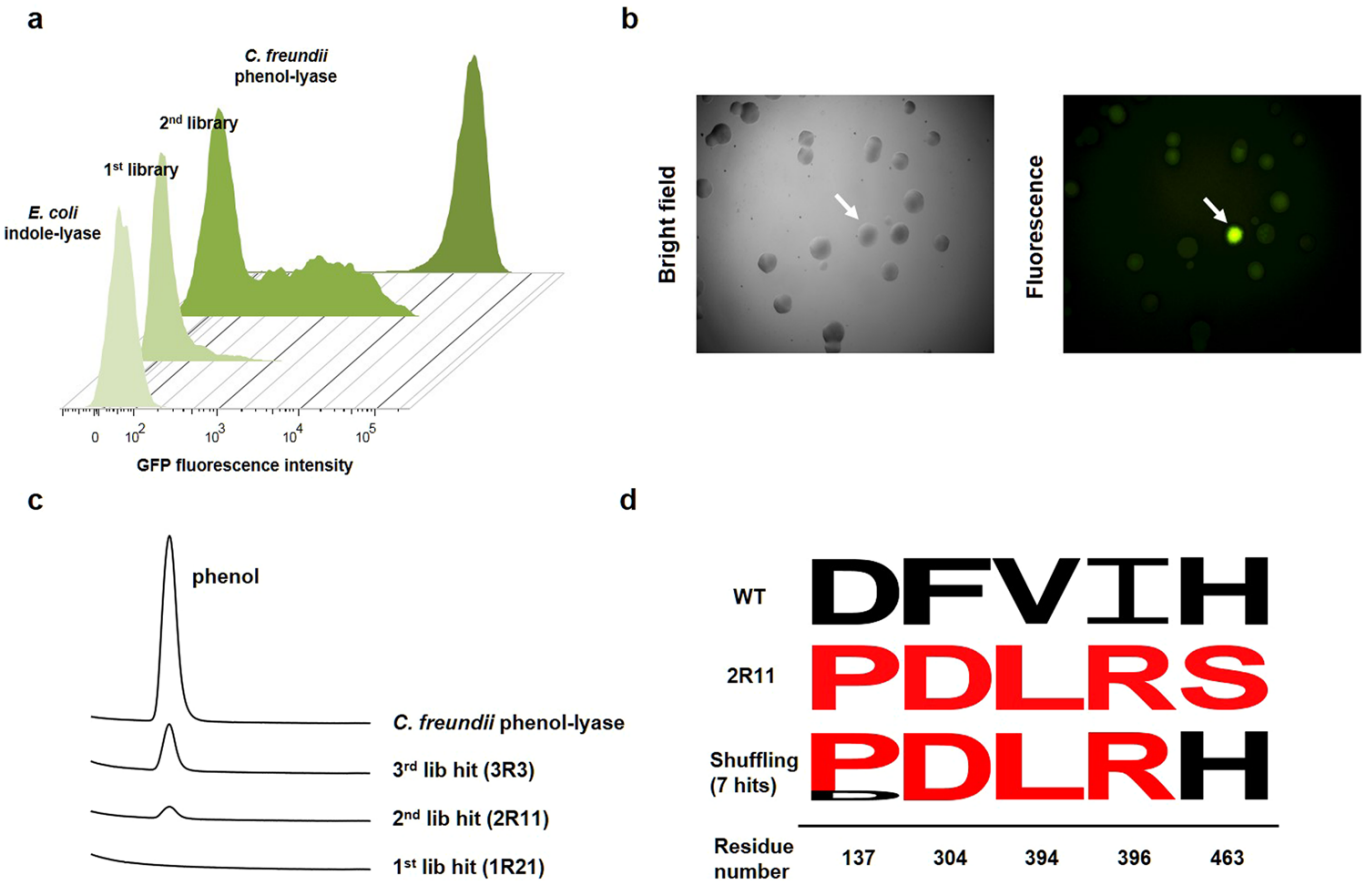
High-throughput screening and development of new phenol-lyases from indole-lyase mutant libraries. (**a**) Fluorescence histograms of the 1^st^ and 2^nd^ mutant libraries obtained using flow cytometry. Cells were grown in M9 broth with 1 mM L-tyrosine. (**b**) Fluorescence image of the 3^rd^ mutant library on M9 agar plate containing l-tyrosine. White arrow indicates the 3R3 variant exhibiting substantial phenol-lyase activity. (**c**) HPLC analysis of products formed by the most beneficial indole-lyase variant sorted from each library. *Citrobacter freundii* phenol-lyase served as the positive control enzyme. (**d**) DNA shuffling of 2R11 phenol-lyase and wild-type indole-lyase. Seven hits from DNA shuffling were analysed using WebLogo to determine the frequency of each mutation. Wild-type indole-lyase residues are shown using black letters, while mutated residues are shown using red letters.

The GESS-based screening procedure involving flow cytometry is summarised in Table 1. Three screening rounds were performed to identify a final TIL variant with significant TPL activity. The histogram of the 1^st^ mutant library showed eighty-one putative hits (Fig. 3a), which were grown using solid and liquid media to confirm the increase in fluorescence. Among them, twenty-one hits showed stronger fluorescence signals than the control cells. However, when analysed by HPLC to detect phenol, none of the twenty-one hits showed a significant phenol peak (Fig. 3c; chromatogram for the 1^st^ library hit). This was probably because the HPLC analysis was not sufficiently sensitive compared to the marginal activity developed in the TIL template. Therefore, the 2^nd^ library was constructed (library size approximately 6 × 10^6^) to collect the beneficial mutations among the eighty-eight mutations present in the twenty-one clones from the 1^st^ library. Eight active variants were isolated by the flow cytometry analysis (Fig. 3a, which depicts a histogram of the 2^nd^ library), followed by colony fluorescence analysis and HPLC. In the HPLC analysis, we identified a 2R11 clone with a significant phenol peak (Fig. 3c, which depicts a chromatogram for the 2^nd^ library hit). The DNA sequence analysis of the 2R11 variant revealed that it harboured six mutations derived from three primary isolates: D137P and F304D from 1R123, V394L and I396R from 1R21 and H463S from 1R157 (Table 1). An unexpected mutation was identified outside the active site, I216M, in the 2^nd^ library. This may have happened during the DNA shuffling process as random mutations can occur at the rate of 1–2 nt per kb^21^. To eliminate detrimental or null mutations in the 2R11 variant, the 3^rd^ library was generated using new DNA shuffling using a 1:1 mixture of the 2R11 variant and WT TIL as the templates. Thirty-two clones with high signals were selected from this shuffling library and activity was confirmed using the colorimetric assay (Fig. 3b). Seven hits with activities higher than that of the 2R11 variant were subjected to sequence analysis and three common mutations (F304D, V394L, and I396R) and the mutation D137P were observed in six of the seven hits (Fig. 3d and Table 1). Finally, we selected the 3R3 variant with D137P, F304D, V394L, and I396R as the template for further analyses.

**Table 1:** Summary of library construction and three screening rounds.

Library	Construction method	Size	Active variants	Major clone(s)	Mutations
First	Saturation mutagenesis	6 × 10^6^	21	1R123	D137P, F304D, I396R
				1R21	D137L, F304S, V394L, I396R
				1R157	D137P, F304S, I396L, H463S
Second	DNA shuffling	6 × 10^6^	8	2R11	D137P, I216M, F304D, V394L, I396R, H463S
Third	DNA shuffling	1 × 10^4^	7	3R3	D137P, F304D, V394L, I396R

### Characterisation of the new phenol-lyase, 3R3 variant

**Figure 4:**
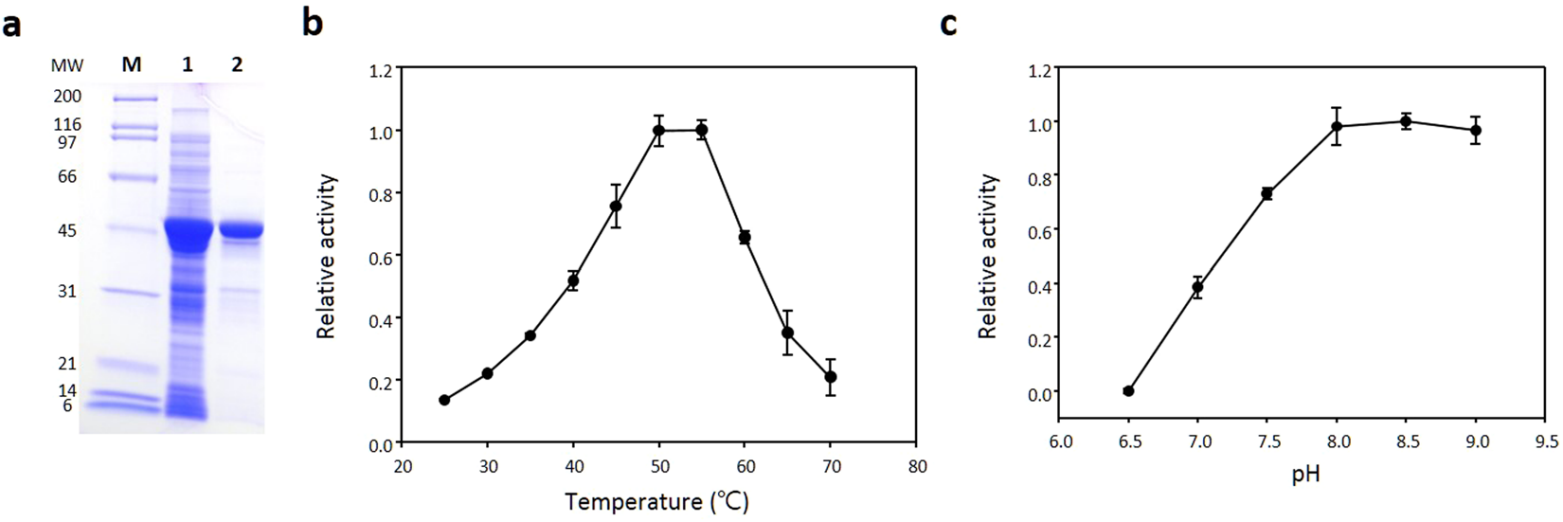
Characterization of synthesised phenol-lyase. (**a**) SDS-PAGE. M: marker; Lane 1: *E. coli* cell extracts expressing the 3R3 variant; Lane 2: purified 3R3 enzyme. (**b**) Effects of temperature on 3R3 phenol-lyase. (**c**) Effect of pH on 3R3 phenol-lyase. All experiments were performed in duplicate.

The 3R3 variant, which differed in four amino acids from *E. coli* TIL, was expressed in *E. coli* JM109 (DE3) Δ*tnaA* strain, purified to homogeneity and characterised for enzymatic properties (Fig. 4a). The maximum activity of the 3R3 variant for the α, β-elimination of l-tyrosine was observed at 50 °C and pH 8.0 (Fig. 4b,c), which was close to that of native TPLs^22–24^. The 3R3 variant displayed a 13-fold lower catalytic efficiency (*k*_*cat*_ /*K*_*M*_ = 0.58 ± 0.05) than that of the wild type (WT) *C. freundii* TPL and 5-fold less l-tyrosine affinity (*K*_*M*_ = 1.18 ± 0.09) (Table 2). Specificity data of 3R3 to other substrates are summarised in Table 3. The 3R3 variant hydrolysed l-tyrosine, l-3,4-dihydroxyphenylalanine, l-serine, and S-ethyl- l-cysteine, but did not hydrolyse the original substrate, l-tryptophan.

**Table 2:** Kinetic parameters of 3R3 phenol-lyase and *C. freundii* phenol-lyase.

Enzyme	*K*_M_ (mM)	*k*_cat_ (sec^−1^)	*k*_ca*t*_/*K*_M_ (mM^−1^ sec^−1)^
3R3 variant	1.18 ± 0.09	0.69 ± 0.03	0.58 ± 0.05
Phenol-lyase^a^	0.24 ± 0.1	1.8 ± 0.2	7.5

^a^From *C. freundii* (Lee, *et al*.^23^).

**Table 3:** Substrate specificity of 3R3 phenol-lyase and wild-type indole-lyase.

Substrate	Relative activity (%)	
	3R3 phenol-lyase	Indole-lyase
l-Tyrosine	100^a^	–^b^
l-Tryptophan	—	100^c^
l-DOPA	20	—
3-Chloro-l-tyrosine	6	—
l-Serine	135	79
S-Ethyl-l-cysteine	200	90

Activity was evaluated using a lactate dehydrogenase-coupled assay to detect pyruvate released from individual substrates (1 mM). ^a^Specific activity: 0.32 U/mg, ^b^Not detected, ^c^Specific activity: 12.4 U/mg.

### Significance of each mutation in 3R3 phenol-lyase

**Figure 5:**
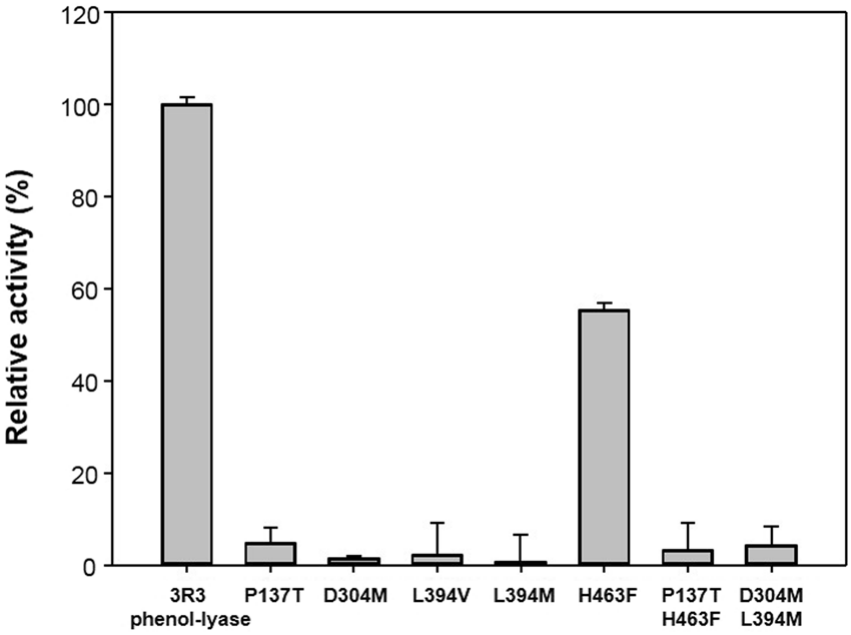
Effect of each mutation on 3R3 phenol-lyase activity. Relative activity of site-directed mutants of 3R3 phenol-lyase. Phenol-lyase activity was monitored for 1 h in 50 mM potassium phosphate buffer containing 50 µM pyridoxal 5-phosphate and 1 mM L-tyrosine. The concentration of generated phenol was examined using a colorimetric method. All experiments were repeated twice.

In the 3R3 variant, the mutations D137P, F304D, V394L, and I396R were reverted to analyse their effects on the new phenol-lyase activity. I396R was not altered because it was presumed to perform the essential function of the base catalyst corresponding to Arg^381^ in the catalytic site of *C. freundii* TPL^12^. Very interestingly, any mutation of the four resides to the conserved amino acids at TIL or TPL abrogated the new phenol-lyase activity, implying that 3R3 activity was closely dependent on all the mutations that occurred (Fig. 2b). Additionally, H463 was not critical to activity, with a 45% reduction in the phenol-lyase activity in the HPLC analysis, explaining the elimination of H463S during the evolution of 3R3. As expected, no combination of single mutations could explain the development of the new enzyme activity (Fig. 5).

## Discussion

High-throughput technologies for quantifying enzyme activities in large mutant libraries have always been a serious concern for enzyme engineering. Here, we introduce a broadly applicable high throughput screening system in conjunction with rational library design as an attempt to create new catalytic activity using a similar scaffold. The library of multiple mutations was expressed in *E. coli* harbouring the genetic biosensor circuit and subjected to flow cytometry. The three screening rounds enriched synergistic mutations and minimised epistatic interactions. The screening process was highly effective in isolating the early hits from numerous genetic variants owing to its extremely high sensitivity and throughput. The early hits showed no significant phenol peaks in the HPLC analysis (Fig. 3a–c). However, they could be bred using the high throughput screening that detects marginal levels of the activities in the genetic library.

The catalytic efficiency of the 3R3 variant of *E. coli* TIL was comparable to that of WT *C. freundii* TPL (7.7% in *k*_*cat*_/*K*_*M*_, Table 2), while the amino acid sequence of 3R3 differed in only four residues. First, Asp^137^ in *E. coli* TIL changed to Pro in 3R3 (Fig. 4a). Asp^137^ is important in the β-elimination of l-tryptophan^25^, while it is conserved as Thr^124^ in most TPLs^26^. Pro^137^ in the active site of 3R3 may possibly contribute to the proper orientation of substrates. The replacement of Pro^137^ with Thr in 3R3 resulted in the complete loss of TPL activity (Figs 5 and S1a). Second, Phe^304^ changed to Asp in 3R3 (Fig. S1b). Phe^304^ is involved in π-π stacking interactions with the substrate of *E. coli* TIL, l-tryptophan. Moreover, the position is related to a conformational change between the open and closed forms, and is conserved as Met^288^ in TPL^27^. However, the replacement of Asp^304^ in 3R3 with Met resulted in no detectable TPL activity (Fig. 5). Third, Val^394^ changed to Leu^394^ in 3R3 (Fig. S1c). When Leu^394^ changed to the consensus residue in TILs (Val) or consensus residue in native TPLs (Met), the 3R3 variant lost most of its activity and negligible levels of phenol were produced, suggesting that Leu^394^ was not replaceable in 3R3. Fourth, Ile^396^ changed to Arg in 3R3 (Fig. S1d). The residue at this position is related to Arg^381^ in WT TPL, which performs an essential function in β-elimination as the base catalyst^12^. Arg^381^ is critical also for the specific binding of substrate at the active site^27^.

**Figure 6:**
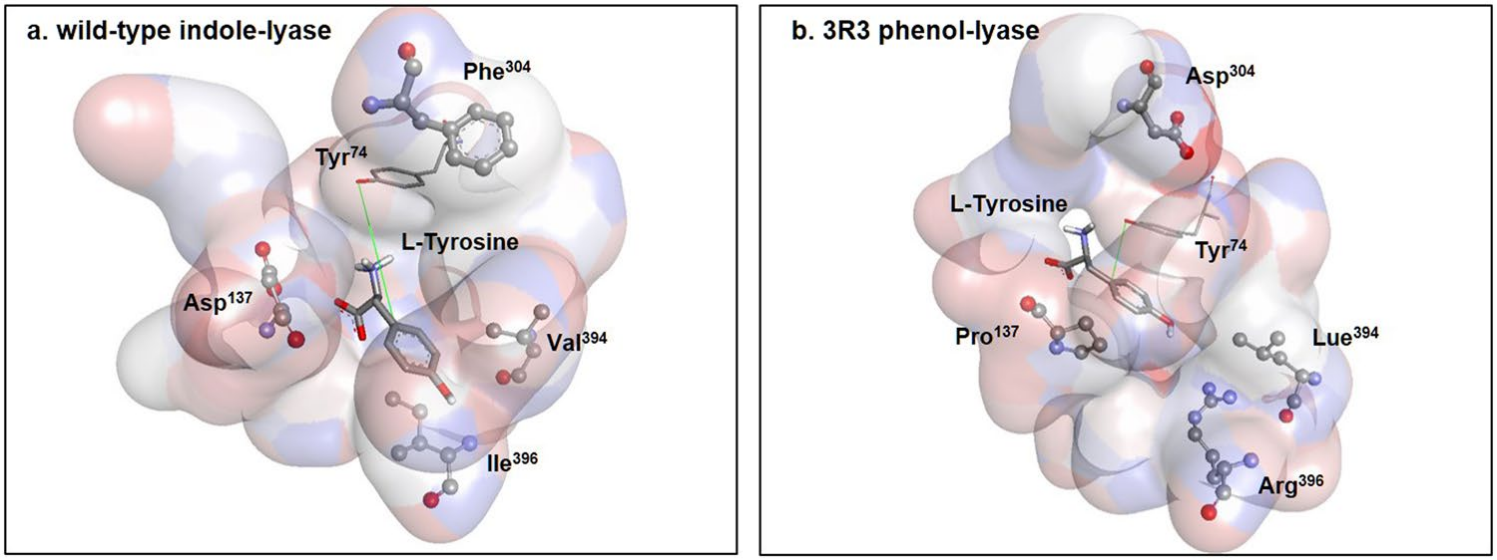
Homology modelling of wild-type indole-lyase and 3R3 phenol-lyase. Both modelled structures were generated using molecular dynamics and docking simulations. The docked ligand and Tyr^74^ are represented as stick and line, whereas the 4 mutated residues are represented as balls and sticks, respectively. The distance between Tyr^74^ and Cγ in the substrate is marked by a green line. The new indole-lyase has a more suitable orientation of the docked ligand regarding atomic distances between the substrate and active site residues.

In addition to the mutational studies, the 3R3 variant was subjected to molecular modelling and ligand docking simulation to determine how the α, β-elimination of l-tyrosine occurred (Fig. 6). In the energy-minimized models of 3R3 and *E. coli* TIL, the remarkable point was the reduced distance of 3.72 Å between the catalytic hydroxyl group of Tyr^74^ and Cγ of the docked l-tyrosine, whereas it was approximately 7.38 Å in the WT model (Fig. 6). Although the free energy of docking with l-tyrosine did not differ significantly between the WT and 3R3 (Table 4), the proximate distance of catalytic residues can be critical to the proper β-elimination reaction of phenol-lyase. When we performed the docking simulation of l-tryptophan into the WT and the 3R3, the docked ligand did not adequately rotate in the active site of the 3R3, whereas the WT displayed proper orientation of docked ligand, which could have been the reason for the loss of the original activity (Fig. S2).

**Table 4:** Chemical parameters of docking simulation of l-tyrosine in 3R3 phenol-lyase and wild type indole lyase.

	3R3 phenol-lyase	Indole-lyase
Docking energy (kcal/mol)	−6.8	−6.6
Distance of Try^74^ from substrate, Cγ (Å)	3.72	7.38

The diversification of five residues was sufficient to create a new phenol-lyase from a related enzyme. The new enzyme comprised unique active site residues to constitute a new TPL activity, which was not replaceable by any residues of the native enzymes. During the test of the 2^nd^ library, an unexpected mutation, I216M, was detected outside the active site (Table 1). This indicates the occurrence of some random mutations during DNA shuffling, which is a recognised event^21^. However, this mutation disappeared automatically during the 3^rd^ DNA shuffling and the high throughput screening. Presumably, the 3R3 variant can be improved further in its catalytic activity after an extensive random mutagenesis or design of a new focused library outside of the active site^28,29^. However, this study focused on the usefulness of hypersensitive genetic screening to create a new active site, rather than focusing on the improvement of TPL activity.

The detection of various enzyme activities using the present biosensor suggests that this method is useful for other activities as well, with the provision of an appropriate substrate with a phenolic group. The limiting factor for this approach is the design of appropriate substrates for the production of phenols and the rational design of the diversifying library from related templates. The approach could also be useful to graft new functions in native cells by merely introducing a few mutations into the homologous gene in the chromosome using precision genome engineering techniques.

This study supports the notion that desired mutations accumulate and deleterious mutations are eliminated rapidly during flow cytometric analysis, favouring changes in the substrate specificity of enzymes. The rapid development of new activity may importantly contribute to biological process development and greater understanding of the relationship between enzyme structure and function.

## Methods

### Materials

All reagents were purchased from Sigma-Aldrich (St. Louis, MO, USA). Restriction endonucleases and T4 DNA ligase were purchased from New England Biolabs (Beverly, MA, USA) and DNA polymerase was obtained from Toyobo (Osaka, Japan). All oligonucleotides were synthesized by Bioneer Co. (Daejeon, Republic of Korea) (Table S1). DNA preparation and manipulation involved standard molecular biological protocols. A miniprep kit (Qiagen, Valencia, CA, USA) was used for plasmid DNA isolation and DNA extraction. The plasmid pGESSv4, consisting of the *dmpR* transcriptional activator, the P_dmp_ promoter from *Pseudomonas putida* KCTC 1452, and the *egfp* gene as the reporter, was used as a genetic circuit system^13^. The genetic circuit was designed to express enhanced green fluorescent protein (EGFP) upon the release of phenol from l-tyrosine through the catalytic activity of an intracellular phenol-lyase. The *tnaA* gene of the *E. coli* host was deleted using a lambda red recombination method^30^ to avoid any interference by endogenous indole-lyase. A kanamycin-resistance cassette was prepared from pKD13 using PCR primers homologous to *E. coli tnaA* regions and removed using pCP20^31^.

### Construction of mutant libraries of indole-lyase

The gene encoding *E. coli* K-12 TIL was cloned into the plasmid pSHCE, which was derived from pHCEIIB (Takara Bio, Shiga, Japan) by replacing the p15A origin of replication from pACYC184, resulting in the generation of pSHCE-Trpase. For constructing the 1^st^ library, the gene encoding *E. coli* TIL in pSHCE-Trpase was amplified by PCR with degenerate primers to diversify target residues (Table S1). The amplified DNA was cloned into pSHCE and the resulting plasmids were transformed into *E. coli* DH5α cells harbouring pGESSv4 to generate the 1^st^ mutant library. All transformants were stored at −80 °C in 15% glycerol solution until daily screening using a flow cytometer. The 2^nd^ library was constructed by DNA shuffling with the Staggered Extension Process (StEP) to recombine beneficial mutations obtained from the 1^st^ library. An equimolar mixture of plasmids selected from the 1^st^ library was amplified by PCR and 2 ng of this mixture was used as the template for staggered extension PCR^21^. The amplified DNA was cloned into the plasmid pRSF (Novagen, Darmstadt, Germany) and the resulting recombinant plasmids were transformed into *E. coli* EPI300 (DE3) harbouring pGESSv4 to yield the 2^nd^ library. The 3^rd^ library was constructed with hits from the 2^nd^ library and WT indole-lyase following the same method and was transformed into *E. coli* JM109 (DE3)*∆tnaA* cells harbouring pGESSv4.

### Library screening using flow cytometry

*E. coli* harbouring the mutant libraries and genetic circuit were screened using a FACSAria™ Flow Cytometer Sorter (BD Biosciences, Franklin Lakes, NJ, USA). The cells were cultured in M9 medium (6.78 g/L Na_2_HPO_4_, 3 g/L KH_2_PO_4_, 1 g/L NaCl, 2 mM MgSO_4_ and 0.1 mM CaCl_2_) supplemented with 0.4% (w/v) glucose, 0.01% (w/v) thiamine, 0.1% (w/v) leucine, 10 µM PLP, 50 µg/mL ampicillin and 25 µg/mL chloramphenicol. After culturing for 15 h at 37 °C, the culture broth was diluted 1:100 with phosphate-buffered saline (PBS) for fluorescence-activated cell sorting (FACS) analysis. A blue laser source (488 nm) and an FL1 (530/30 nm) photomultiplier tube were used to analyse EGFP fluorescence. Forward and side scatter were measured to exclude debris and dead cells. In total, 10^7^ cells were counted for each library over 30 min. Data were acquired using BD CellQuest Pro (version 4.0.2, BD Biosciences) and analysed using Flowjo software (Flowjo, Ashland, OR, USA). The top 0.1% of cells with the highest fluorescence were sorted directly into 1 mL of Luria-Bertani (LB) medium (histogram for 1^st^ library is presented in Fig. 3a) and grown on 10 M9 agar plates containing 1 mM L-tyrosine for further screening.

### Solid and liquid-phase fluorescence analyses

Imaging of fluorescent colonies was performed using an AZ100M fluorescence Multizoom microscope (Nikon, Tokyo, Japan) equipped with a GFP filter (excitation at 455–485 nm, emission at 500–545 nm). After 48 h of incubation on M9 agar plates at 37 °C, the fluorescence of the developed colonies was observed and 100 colonies displaying fluorescence higher than that of the control cells were transferred to M9 broth containing 1 mM L-tyrosine. Fluorescence intensity was measured after 15 h at 37 °C using a multi-label plate reader (PerkinElmer, Waltham, MA, USA) at excitation and emission wavelengths of 485 nm and 535 nm, respectively.

### Expression and purification of enzyme variants

Genes encoding active TIL variants and WT indole-lyase were cloned into the *Nco*I/*EcoR*V restriction site of pETDuet-1 (Novagen) and expressed in *E. coli* JM109 (DE3) ∆*tnaA*. The recombinant cells were cultured in LB medium at 37 °C in a shaking incubator and isopropyl β-D-1-thiogalactopyranoside (IPTG) was added at a final concentration of 0.5 mM, when the optical density at 600 nm (OD_600_) reached 0.4 and the cells were further incubated for 6 h. The cells were harvested by centrifugation at 5,000 rpm for 10 min. The pellet was resuspended in lysis buffer containing 50 mM sodium monophosphate, 300 mM NaCl, 10 mM imidazole and 0.1 mM phenylmethylsulfonyl fluoride as protease inhibitor, and disrupted by sonication for 3 min on ice. The lysate was centrifuged at 15,000 rpm for 10 min to remove cell debris. The supernatant was used directly to purify the His-tagged enzymes by loading into a Profinia™ Affinity Chromatography Protein Purification System (Bio-Rad, Hercules, CA, USA). The purified enzymes were confirmed by sodium dodecyl sulphate polyacrylamide gel electrophoresis (SDS-PAGE) and protein concentrations were quantified by the Bradford method. The purified enzymes were immediately examined for biochemical and kinetic characterisation.

### Enzyme assay and HPLC analysis

Phenol-lyase activity was monitored for 1 h in 50 mM potassium phosphate buffer containing 50 µM PLP and 1 mM l-tyrosine. The concentration of the generated phenol was analysed using a HPLC system (Shimadzu, Kyoto, Japan) equipped with a C18 reversed-phase column (Chemco, Coatbridge, UK) and Chromopak UV detector (detection at 268 nm). The mobile phase consisted of 50% acetonitrile and 50% water at a flow rate of 1 mL/min. Additionally, the concentration of phenol in the reaction mixture was determined using a colorimetric method in which 100 µL of the enzyme assay solution was added to 100 µL of 0.1 M NaOH, 30 µL of 0.6% (w/v) 4-aminoantipyrine and 30 µL of 0.6% (w/v) potassium persulphate, and incubated for 10 min to evaluate the colour change using a well plate reader^32^. One unit (U) of the enzyme with l-tyrosine as the substrate was defined as the amount required to liberate 1 μmol phenol per minute at 50 °C and pH 8.0.

### Protein-structure modelling and docking simulations

The structure of WT indole-lyase is based on its X-ray crystallographic structure with the Protein Data Bank (PDB) ID, 2C44. Indole-lyase is a tetramer with four chains, namely A, B, C, and D. Two neighbouring chains (A/B or C/D dimers) are directly involved in chemical reactions. The dimer of A and B chains was obtained from PDB for initial structure building. With the dimer, short energy minimization and molecular dynamics (MD) simulation analyses were performed to relax the protein structure. The backbone atoms were constrained to their original atomic coordinates using harmonic potential. The force constant of the harmonic potential gradually decreased from 1, 0.5, and 0.0 kcal/mol as MD simulation progressed. The initial structure of the 3R3 variant was based on the structure of WT indole-lyase. For initial structure building for the 3R3 variant, common atomic positions, such as backbone, Cβ atoms and so on, from WT indole-lyase were maintained. The remaining missing atoms (D137P, F304D, V394L, and I396R) were generated using internal coordinate manipulation from Chemistry at HARvard Macromolecular Mechanics (CHARMM)^33^. The same protocol was used for the generation of the structure of WT indole-lyase. After MD simulations, two relaxed structures were obtained and they were used for docking simulation by AutoDock Vina with l-tyrosine and l-tryptophan as ligands^34^. The pocket residues for docking were identified using the pocket detection programme Pck (http://schwarz.benjamin.free.fr/Work/Pck/home.htm). With the detected pocket residues, (296 residues detected), in total 2,960 docking simulations were performed involving ten attempts of docking simulations for each pocket residue with different random seeds. The 2,960 docked ligand poses were clustered and grouped. Clustering was based on the centres of mass (COMs) of the docked ligands. The group with the lowest energy conformation was chosen for further analyses. The chemical activities of WT and mutant indole-lyase were quantified in terms of the docking energy (unit being kcal/mol) of the ligands and their orientation within the binding pocket. The appropriate orientation of tyrosine was expressed in the atomic distance between tyrosine and active site residues.

## Electronic supplementary material

supplementary information
