# Supplementary material for: Evolution of enzymes with new specificity by high-throughput screening using DmpR-based genetic circuits and multiple flow cytometry rounds
Source: Sci Rep. 2018 Feb 8;8:2659. doi: 10.1038/s41598-018-20943-8 (PMC5805759; doi:10.1038/s41598-018-20943-8)

*Supplementary Information*

**Evolution of enzymes with new specificity by high-throughput screening using DmpR-based genetic circuits and multiple flow cytometry rounds**

Kil Koang Kwon^1,2^, Dae-Hee Lee^1,3^, Su Jin Kim^1^, Su-Lim Choi^1^, Eugene Rha^1^, Soo-Jin Yeom^1^, Bindu-Subhadra^1^, Jinhyuk Lee^4,5^, Ki Jun Jeong^2*^ and Seung-Goo Lee^1,3*^

**Table S1**. Oligonucleotides used in this study

| Primer | Sequence (5´to 3´) ^a^ | Remark |
| --- | --- | --- |
| tna-SAT-F1 | TAAATTGAATTTTCACACTTCTGGAAAAAGGAGATATCATATGGA AAACTTTAAACATCTCCC | Generation of 1^st^ library |
| tna-SAT-R1 | GCACGGTACAGCCGTTGATCTGGCTATGGCCCTGCGTGGTNNNAA AGAAATAGTTAGAGAACGCC |
| tna-SAT-F2 | GGATCGCAGCAAAATGGTGGCGTTCTCTAACTATTTCTTTNNNAC CACGCAGGGCCATAGC |
| tna-SAT-R3 | GACGCTCCATCGCGCCGCCTTCTAGGCCGCCATATGTCGGNN NGCCTTCCTGCACCACGC |
| tna-SAT-F4 | GTACACCGAGTGCAGAACCCTTTGCGTGGTGCAGGAAGGCNN NCCGACATATGGCGGCCTA |
| tna-SAT-R4 | GTTTACCGGTTTTCGGATCGCGGCCTAACAGGAAAGAGCCNN NTTCNNNCGCACGGATACCG |
| tna-SAT-F5 | CTGCGAGCTGTATAAAGTCGCCGGTATCCGTGCGNNNGAANN NGGCTCTTTCCTGTTAGGCC |
| tna-SAT-R5 | CGCCAAAACAGCCAAGCTTAAACTTCTTTAAGTTTTGCGGTGAAN NNACGCAATACTTTCGGTTCGT |
| tna-F1 | CAATTCCCCTGTAGAAATAATTTTGTTTAACTTTAATAAGGAGAT ATACCATGGAAAACTTTAAACATCTCCCTG | Generation of 2^nd^ and 3^rd^ library |
| tna-R1 | AGACTCGAGGGTACCGACGTCAGCGATCGCGTGGCCGCCCGATATCTCAGTGGTGATGATGGTGATGAACTTCTTTAAGTTTTGCGGTGA |
| tnaA-del1 | TGTAGGGTAAGAGAGTGGCTAACATCCTTATAGCCACTCTGTAGTATTAAGTGTAGGCTGGAGCTGCTTCG | Knock-out of *E. coli* *tnaA* |
| tnaA-del2 | CAGATGTAATATTCACAGGGATCACTGTAATTAAAATAAATGAAGGATTATGTAATTCCGGGGATCCGTCGACC |

^a^ The restriction sites in the nucleotides are underlined.

**Figure S1**. The aligned structures of 3R3 phenol-lyase and tryptophan indole-lyase. The backbone and four mutations in the 3R3 homology model were represented in yellow lines and green sticks, respectively, and the corresponding four residues in tryptophan indole-lyase were marked in pink. The substrate tyrosine in the 3R3 model was positioned by docking using Autodock vina and was represented in grey. **a**. Pro^137^ in the homology model, **b**. Asp^304^ in the homology model, **c**. Leu^394^ in the homology model, **d**. Arg^396^ in the homology model. The distance between oxygen in the substrate and Arg^396^ in 3R3 was marked by a green line.


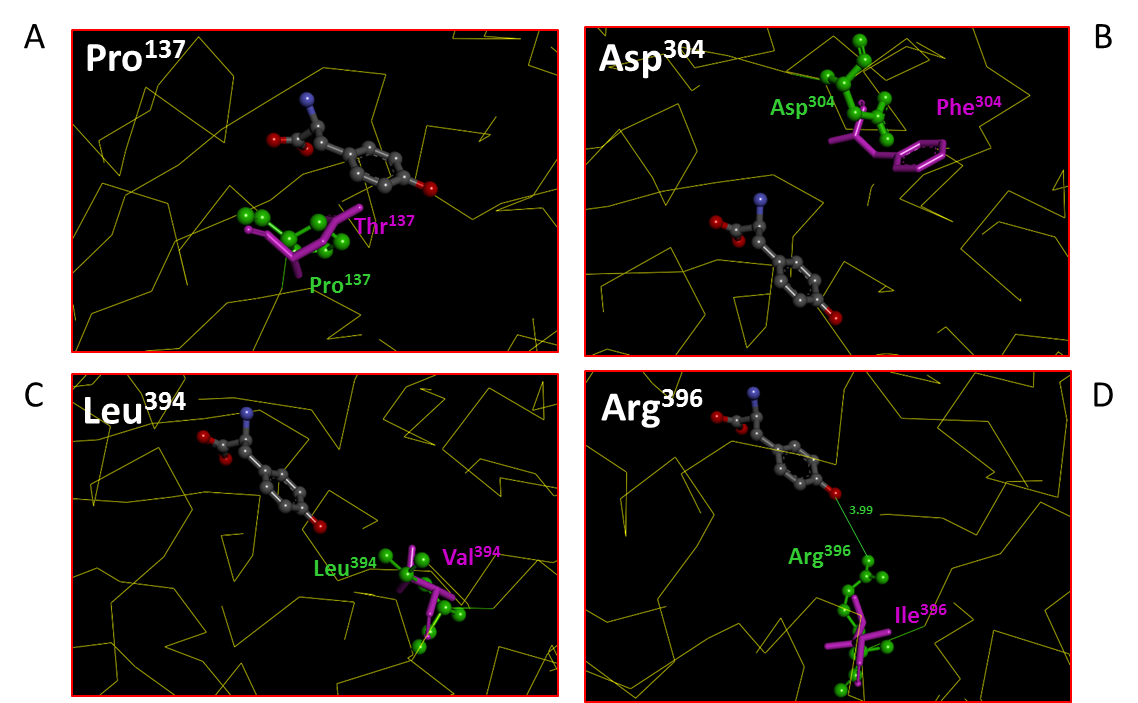


**Figure S2**. Docking simulation of l-tryptophan into wild-type and new indole-lyase. The docked ligand and Tyr^74^ were represented as sticks, while 4 mutated resides represented as ball and stick. The wild-type structure has suitable orientation of docked ligand in terms of the distance between substrate indole ring and Asp^137^ whereas the 3R3 has impractical orientation.


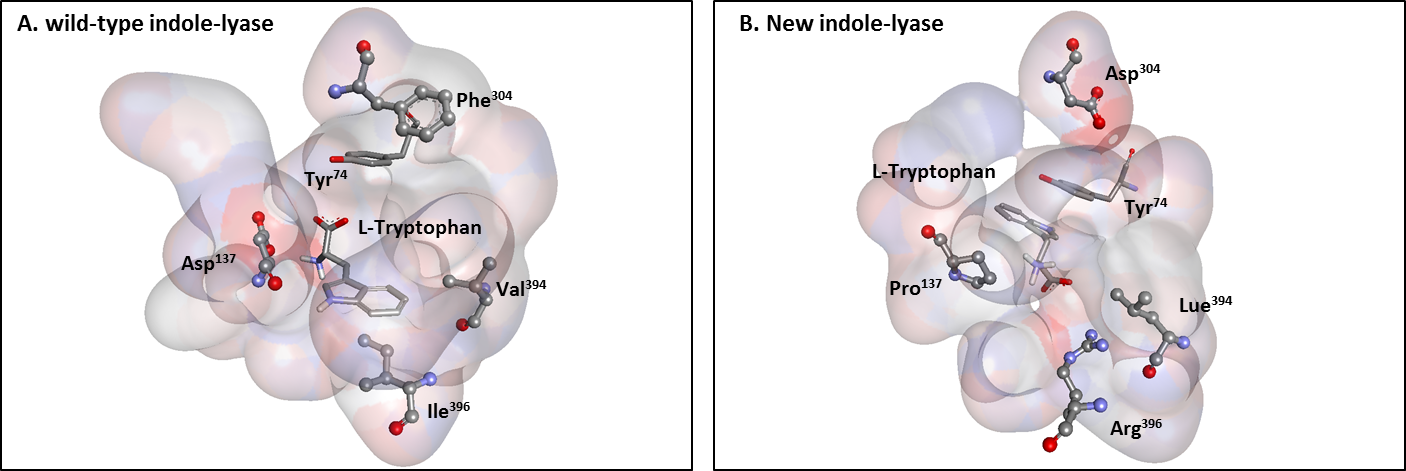

Supplement: Supplementary file 1 — supplementary information [file 41598_2018_20943_MOESM1_ESM.doc]
